# Inconsistent kinetic isotope effect in ammonia charge exchange reaction measured in a Coulomb crystal and in a selected-ion flow tube

**DOI:** 10.1038/s41467-022-30566-3

**Published:** 2022-06-09

**Authors:** Shaun G. Ard, Albert A. Viggiano, Brendan C. Sweeny, Bryan Long, Nicholas S. Shuman

**Affiliations:** 1 Air Force Research Laboratory, Space Vehicles Directorate, Kirtland Air Force Base, Albuquerque, NM 87117 USA; 2 Institute for Scientific Research, Boston College, Boston, MA 02467 USA; 3 NRC Postdoc at Air Force Research Laboratory, Space Vehicles Directorate, Kirtland Air Force Base, Albuquerque, NM 87117 USA

**Keywords:** Reaction kinetics and dynamics, Chemical physics

**arising from** L. S. Petralia et al. *Nature Communications* 10.1038/s41467-019-13976-8 (2020)

The recent paper by Petralia et al.^1^ reports a strong inverse kinetic isotope effect (KIE) of 0.3 ± 0.05 between the reactions of Xe^+^ (^2^P_3/2_) with NH_3_ and ND_3_. The measurement was taken with the Xe^+^ sympathetically cooled by Ca^+^ atoms in a Coulomb crystal to ~30 K and the NH_3_/ND_3_ introduced ambiently at 290 K. The authors point out that the observed KIE was unusual and speculate on why it might occur. We have measured the temperature-dependent kinetics (175–600 K) of both reactions using a variable temperature selected-ion flow tube (SIFT), reporting a KIE near 1 for all temperatures. The current rate constants agree reasonably well with the Coulomb crystal measurements for both the NH_3_ and ND_3_ reactions given the absolute uncertainties. However, the differences are in opposite directions resulting in a large discrepancy in the KIE between the experiments.

Measurements were performed using the previously described variable ion source and temperature-adjustable selected-ion flow tube (VISTA-SIFT)^2^. Ions were produced using an electron impact ion source yielding both ground Xe^+^(^2^P_3/2_) and excited state Xe^+^(^2^P_1/2_), then mass-selected and injected into a flow tube maintained at ~0.3 Torr of fast flowing helium gas. In order to ensure that the present results refer only to Xe^+^(^2^P_3/2_), we used the method of Smith and Adams^3,4^ to selectively react Xe^+^(^2^P_1/2_) with N_2_O upstream in the flow tube prior to the addition of NH_3_/ND_3_. The rate constants were measured by monitoring the first order decay of Xe^+^(^2^P_3/2_) as a function of the concentration of NH_3_/ND_3_. Without N_2_O present, the Xe^+^ signal decayed via a double exponential indicating the reactive contribution of both states. With N_2_O added, single exponential decay was observed over three orders of magnitude, indicating >99.9% of ions were in the ground state. Temperature was varied by either resistively heating the entirety of the flow tube or by pulsing liquid nitrogen through copper tubing braised to the flow tube. Collisions between the reactant species and the helium buffer gas ensure rapid thermalization to the temperature of the flow tube wall. Errors in these measurements are +/−30% absolute and +/−20% relative, as is typical for this technique^5,6^. Reaction with contaminants or isotopic mixing of NH_3_ and ND_3_ are ruled out as time-of-flight mass spectra are taken for the entirety of the experiments and the only product ions observed are clearly identified and directly attributed to primary or secondary chemistry with the supplied reactants. The rate constants reported are directly determined from Xe^+^(^2^P_3/2_) signal decrease, but are consistent with those derived from unquenched data including Xe^+^(^2^P_1/2_), as well as from modeling the product ion increases observed, but only when properly accounting the continued chemistry of the product ions with NH_3/_ND_3_ (e.g., NH_3_^+^ + NH_3_ → NH_4_^+^ + NH_2_)^7^.

Figure 1 shows the rate constants measured as a function of temperature for both reactions and Fig. 2 shows the KIE (*k*_NH3_/*k*_ND3_) as a function of temperature. Both reactions occur at an appreciable fraction of the Su-Chesnavich parameterized collision rate^8^ for ion-permanent dipole reactions. The Coulomb trap experiments were estimated to be at an effective translational temperature of ~260 K. For the ND_3_ rate constants, the agreement is reasonable, within the error of either measurement. The agreement for the NH_3_ reaction is marginal, only within mutual uncertainty of both measurements, and the difference is opposite that of ND_3_. The present results agree well with three previous room temperature values for the NH_3_ reaction including two performed at low pressure^3,4,9,10^. For the KIE, the agreement is not satisfactory, with the present measurements consistent with a value of 0.95 ± 0.15 at all temperatures with a possible small positive temperature dependence, while the Coulomb crystal value is 0.3 ± 0.05 at 260 K.

**Fig. 1:**
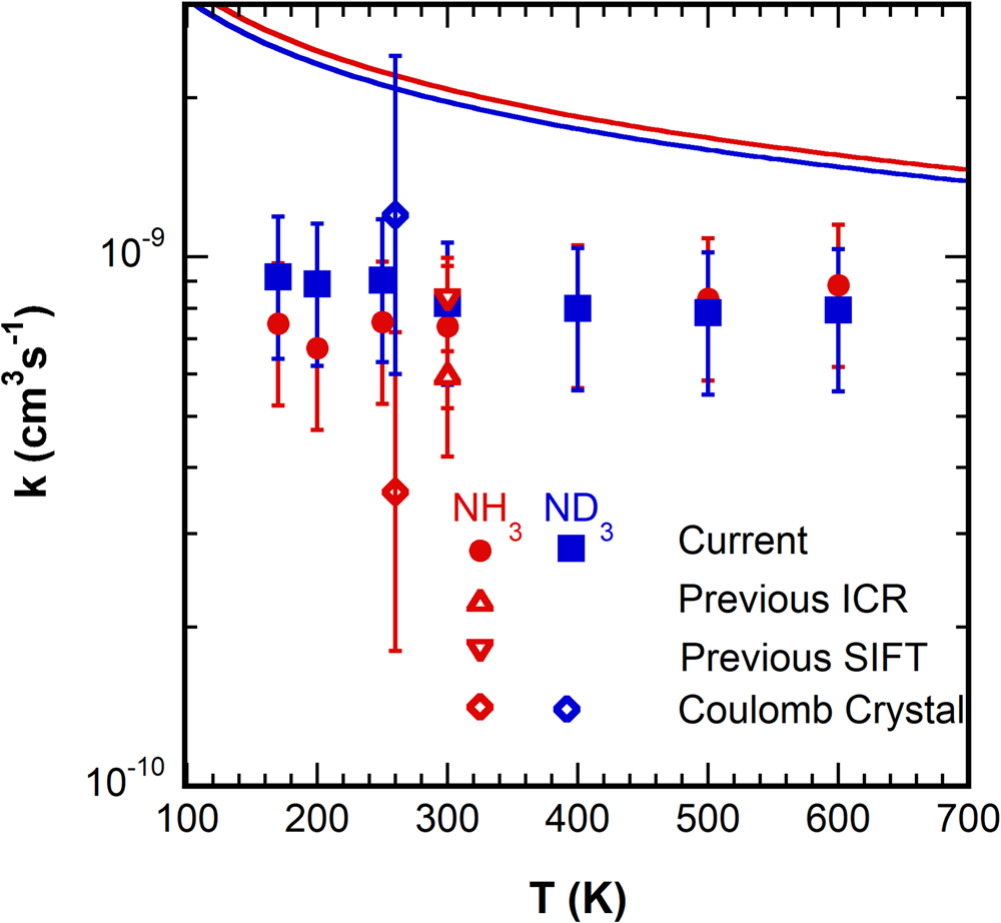
Comparison of Xe^+^ + NH_3_/ND_3_ rate constants. Experimental rate constants for Xe^+^ + NH_3_(red)/ND_3_(blue) as a function of temperature for the present work (solid circles/squares) with error bars indicating the 95% confidence region, as well as those previously published (open symbols): Derai et al. (up triangle, ref. ^9^) and Chau and Bowers (up triangle, ref. ^10^), Smith, Adams and co-workers (down triangle, ref. ^3,4^), Petralia et al. (diamonds, ref. ^1^). Solid curves are the parameterized capture rate constants from ref. ^8^.

**Fig. 2:**
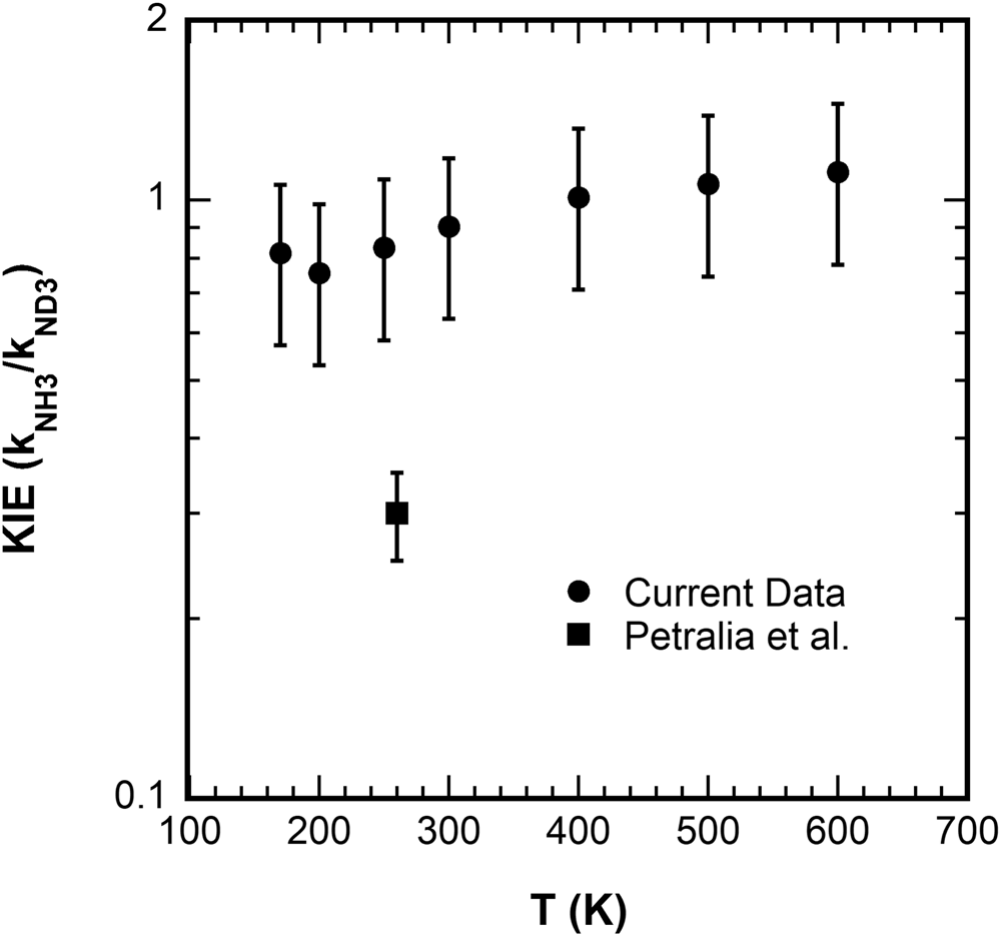
Comparison of kinetic isotope effects for Xe^+^ + NH_3_/ND_3_. Experimentally determined kinetic isotope effect (*k*_(Xe+ + NH3)_/*k*_(Xe+ + ND3)_) as a function of temperature derived from the present work (circles) and of Petralia et al. (ref. ^1^) squares. Error bars indicate the 95% confidence region.

It should be noted that the experiments take place under very different pressure regimes: ~0.3 Torr in the current work, as well as that by Smith, Adams, and co-workers^3,4^, ~5 × 10^−6^ Torr in the ICR work of Derai et al. and of Chau and Bowers^9,10^, and ~4 × 10^−9^ Torr in the work of Petralia et al.^1^. A pressure effect would require the lifetime of a (Xe-NH_3_/ND_3_)*^+^ complex to be long enough such that a collision with the helium buffer occurs (~200 ns under the SIFT conditions). The complex lifetimes were calculated using statistical theory (implemented using the simplified statistical adiabatic channel model^11^ using molecular frequencies calculated at the MP2/def2-TZVP level) to be much shorter, between 50 and 500 ps across the relevant temperature range assuming the extremes of statistical theory, i.e., phase space theory (PST) or Rice–Ramsperger–Kassel–Marcus (RRKM) theory. In either limit, it is unlikely for the complex to encounter even a single collision with helium. The pressure dependence for the NH_3_ reaction was measured using the SIFT over the modest accessible range of 0.2–0.6 Torr and no dependence in the rate constant was found. While we cannot definitively rule out pressure as a cause of the discrepancy, it appears unlikely.

## Data Availability

The data supporting the finding of this study will be available from the authors upon request.

## Code availability

Statistical modeling code used in this study will be available from N.S.S. upon request.
